# Assess the Association Between Periodontitis and Maxillary Sinusitis: A Cross-Sectional Cone-Beam Computerized Tomography (CBCT) Study

**DOI:** 10.7759/cureus.48587

**Published:** 2023-11-09

**Authors:** Raed M AlRowis, Adel H Alzahrani, Saud H Alzuhair, Khaled A Almalhook, Abdelaziz W Almasry, Hebah M Hamdan

**Affiliations:** 1 Periodontics and Community Dentistry, College of Dentistry, King Saud University, Riyadh, SAU; 2 Oral Medicine and Diagnosis Sciences, College of Dentistry, King Saud University, Riyadh, SAU; 3 Dentistry, College of Dentistry, King Saud University, Riyadh, SAU

**Keywords:** cross-sectional study, cone-beam computed tomography, maxillary sinus mucosal thickening, alveolar bone loss, maxillary sinusitis, periodontitis

## Abstract

Background/purpose

Periodontal pathologies which are considered odontogenic in origin can be a major cause of maxillary sinusitis, along with other dental and non-dental causes. The aim of this study is to define and assess the relationship between periodontitis and maxillary sinusitis.

Material and methods

A total of 415 CBCT datasets of periodontitis patients were cross-sectionally evaluated. Alveolar bone loss and maxillary sinus mucosal thickening were measured in coronal and sagittal sections, these two variables represent the severity of periodontitis and maxillary sinusitis, respectively.

Results

This study found that mucosal thickening was significantly higher in patients with increased alveolar bone loss severity (P=0.03). Mucosal thickening was significantly higher among males (83.5%) than among females (69.8%) (P=0.001). moderate or severe alveolar bone loss had a significantly higher risk of mucosal thickening with an odds ratio of 1.8 when compared to those with mild alveolar bone loss (95% CI: 1.04-3.2). Males had an increased risk of mucosal thickening compared to females with an odds ratio of 2.2 (95% C.I.: 1.4-3.6).

Conclusion

In conclusion, periodontal structure can affect maxillary sinus and its health. Therefore, after confirming a diagnosis of maxillary sinusitis, a detailed examination of periodontal health is needed. These results can be used to increase the awareness of dental students and practitioners in clinical and diagnostical judgement.

## Introduction

Periodontitis is defined by pathologic loss of the periodontal ligament and alveolar bone [1,2]. Periodontal diseases impact between 20% and 50% of the global population in both industrialized and developing countries affecting adolescents, adults, and the elderly [2]. In this context, a dental issue underlies close to one-third of cases of unilateral maxillary sinusitis [3]. Maxillary sinusitis is defined as symptomatic inflammation of the maxillary sinus and is classified as chronic when it lasts longer than 12 weeks. The origin of sinusitis is thought to be primarily rhinogenous, but in some instances, dental infection is a major predisposing factor [4-7]. Furthermore, both periodontitis and odontogenic sinusitis (OS) are multifactorial and polymicrobial infections [1,2]. OS accounts for 10%-12% of all sinusitis cases, although recent findings suggest that it could be as high as 41% [5].

OS is underappreciated in the current literature [8,9]. Diagnosing this disease can be challenging for the practitioner [8]. While symptoms of odontogenic and non-OS are similar, it increases the chance of misdiagnosis [9]. An accurate diagnosis of odontogenic origin is a must for appropriate management [5]. Multiple causes contribute to the disease, but one of the most frequent is periodontitis [6,10]. Teeth with roots within the maxillary sinus were about twice as likely to be related to maxillary sinus pathologies [10]. Anatomically, maxillary posterior teeth are close to the maxillary sinus, which also shares a blood supply, which may explain why inflammation is transmitted to the sinus from periodontal pockets [11-13]. According to Roque-Torresa et al., 83% of OS is caused by apical periodontitis and other periodontal disorders [10]. Due to the close anatomical relationship between the maxillary posterior teeth and the maxillary sinus, the chance of sinus inflammation with an odontogenic cause would rise [14-17]. This close relationship has been a constant challenge for practitioners, while peri-radicular and periodontal pathologies originating from posterior maxillary teeth may spread into the sinus [14]. Periodontal diseases are caused by several factors. The most significant risk factors for periodontitis are smoking, diabetes mellitus, and poor oral hygiene [2]. These modifiable and non-modifiable risk factors contribute to the clinical importance of periodontal disease [2].

The literature has revealed a clear correlation between periodontal disease and OS [15-23]. Several known causes of OS include iatrogenic extrusion of foreign bodies into the sinus, apical periodontitis, and periodontal disease [6]. Periodontitis is considered the most common cause of OS [24]. These conditions may result from advanced dental disease; the mucosal lining of the maxillary sinus often thickens as a first reaction to these kinds of conditions, followed by bacterial infections and periodontal pathologies, which can lead to alveolar bone loss (ABL) [25-28]. Maxillary sinus mucosal thickness is significantly correlated with periodontal bone loss. Mucosal thickening (MT) was three times more frequent in the maxillary sinuses of individuals with severe periodontal bone loss [25]. The maxillary sinus is completely lined by the Schneiderian membrane, a thin respiratory membrane of mucus that is strictly attached to the periosteum. On radiographic assessment, healthy maxillary sinus mucosa may not be visible or its thickness may be within 2 mm. MT > 2 mm can be classified as pathological [23].

Localized MT is more commonly linked to periapical lesions, whereas generalized MT is more frequently linked to periodontal bone loss [27-30]. If the underlying odontogenic cause of sinus inflammation is not detected, management will not be efficient [15]. The inflamed sinuses are directly proportional to the severity of the periodontal disease [18,28,30].

Cone-beam computerized tomography (CBCT) provides higher-resolution isotropic volume data and is advantageous for examining the bony characteristics of the maxillary sinus by applying a lower dose in comparison to computerized tomography [31]. The maxillary sinus is implicated in several fields of dentistry, and assessing it is essential for the appropriate management and detection of maxillary sinus pathologies [31-35]. CBCT is considered a gold standard for the diagnosis of sinusitis [23,26]. 2-D imaging models such as panoramic, peri-apical, and bite-wings have limitations in the diagnosis of sinusitis. In contrast, CBCT has high spatial resolution and higher accuracy in detecting apical periodontitis and MT [34,36,37].

The significance of this study is to facilitate the recognition of the early association between periodontitis and maxillary sinusitis, raise awareness among dental students, general practitioners, and specialists, and reinforce educational materials in periodontology and ENT. Moreover, the objective of this study was to define the relationship between and probability of occurrence of these two diseases. This study investigates periodontitis patients and their maxillary sinus health in a population that has not been explored which fills a gap in the literature.

## Materials and methods

CBCT image selection

The study protocol was in full agreement with the Scientific Research Ethics of the Institutional Review Board (IRB) of King Saud University (approval No. E-22-7170). Necessary measures were taken to protect patient privacy during data collection, analysis, and publication.

Patients were selected from the periodontics department at the Dental University Hospital of King Saud University using the Salud software system (Dental Management and Organization system). All periodontitis cases were diagnosed based on radiographic parameters (bone loss) and cross-sectionally evaluated between November 2022 and June 2023. The confirmation of diagnosis was done by reviewing the progress notes of each case selected. 

The inclusion criteria were as follows: (1) patients who had been diagnosed with periodontitis; (2) CBCT images that clearly showed at least one maxillary sinus and associated posterior maxillary teeth; and (3) patients older than 18 years. The exclusion criteria were: (1) edentulous patients, that is, missing posterior maxillary teeth; (2) patients diagnosed with rheumatoid arthritis; (3) patients diagnosed with osteoporosis; (4) patients diagnosed with systemic lupus erythematosus; (5) patients with implants in the posterior maxilla; and (6) periapical lesions such as apical periodontitis and/or periapical abscess.

Data collected

**Figure 1:**
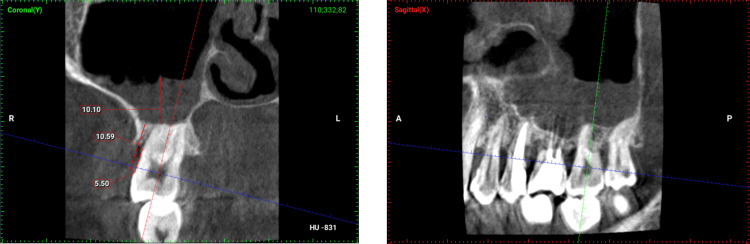
Coronal and sagittal sections of CBCT radiograph with measurements (R) right side, (L) left side, (A) anterior, (P) posterior CBCT – cone-beam computerized tomography

Two examiners evaluated 415 CBCT datasets of periodontitis patients treated at the Dental University Hospital at King Saud University Medical City. The examiner was a dental intern who had undergone training by a board-certified oral and maxillofacial radiologist with 11 years of experience. The mucosal thickness and periodontal bone loss were assessed in corrected coronal and sagittal views along the long axis of the tooth using Planmeca Romexis® software (version 5.2.0.R, Planmeca, Helsinki, Finland). Mucosal thickness was measured using a line drawn from the boundary of the mucosal lining to the floor of the maxillary sinus at a 90° angle (Figure 1).

Study variables included ABL, maxillary sinus MT, age, sex, nationality, and medical history. Bone loss was measured on CBCTs using the cemento-enamel junction as a reference, and bone loss was calculated as a percentage of the total length of the root (Figure 1). However, according to Zhang et al., an ABL equal to or less than 2 mm is considered normal since it is the biological width [28]. Moreover, ABL is classified as follows: Mild = <25% bone loss; moderate = 25-50% bone loss; severe = >50% bone loss [38].

The study cases were classified according to MT as a binary variable (yes/no). According to Roque-Torresa et al., a slight MT of less than 2 mm is a common finding. However, a thickening > 2 mm can be considered a sign of maxillary sinusitis [10].

Comparing the two examiners using Kappa’s agreement, the first examiner’s result was 0.449, which was approximately (0.45), while the second examiner’s result was 0.4454, which was approximately (0.45). The correlation between the two examiners was very strong (0.969), indicating a high level of agreement.

Statistical analysis

All data were analyzed using SAS software, version 9.4 (SAS Institute Inc., Cary, NC, USA). Descriptive statistics, including means ± standard deviation, were computed for continuous variables, while frequencies and percentages were computed for categorical variables. To examine whether ABL and other background variables (including age, gender, nationality, and presence of medical condition) were associated with maxillary sinus MT, bivariate analyses were conducted using chi-square and Fisher’s exact tests. Variables found to be significant from bivariate analyses or reported to be key determinants for MT in literature were considered for entry into the final multivariate logistic regression model. The final model was evaluated for its fit using the Hosmer-Lemeshow goodness-of-fit test. Our analysis demonstrated that the model had a satisfactory fit. Statistical differences with a P < 0.05 and confidence intervals of 95% were considered to be significant.

## Results

As shown in Table 1, a total of 415 cases were included in the study, all of which were periodontitis cases; females were slightly higher with 215 cases (51.8%) in comparison to 200 males (48.2%). Regarding nationality, only 35 cases were non-Saudi, with the rest being Saudi cases; 102 cases had a systemic disease, and others were medically fit.

**Table 1:** Background characteristics of the study participants (n=415) * Statistically significant ‡ABL: Mild = Bone resorption < 25%, Moderate = Bone resorption between 25% and 50%, Severe = Bone resorption > 51%

		MT		
	Total sample	No	Yes	P-value
Overall N (%)	415 (100)	98 (23.6)	317 (76.4)	
Age N (%)				
18-35 years	116 (28.0)	29(25.0)	87(75.0)	0.6
36-49 years	134 (32.3)	27(20.2)	107(79.9)	
50-59 years	87 (21.0)	23(26.4)	64(73.6)	
≥60 years	78 (18.8)	19(24.4)	59(75.6)	
Gender N (%)				
Male	200 (48.2)	33(16.5)	167(83.5)	0.001*
Female	215 (51.8)	65(30.2)	150(69.8)	
Nationality N (%)				
Saudi	380 (91.6)	94(24.7)	286(75.3)	0.09
Non-Saudi	35 (8.4)	4(11.4)	31(88.6)	
Presence of medical condition N (%)				
Yes	102 (24.6)	29(28.4)	73(71.6)	0.2
No	313 (75.4)	69(22.0)	244(78.0)	
ABL N (%)‡				
Mild	83 (20.0)	27 (32.5)	56 (67.5)	0.03*
Moderate/Severe	332 (80.0)	71 (21.4)	261 (78.6)	

Among participants, 28% (n=116) were aged between 18 and 35 years old, 32.3% (n=134) were aged 36-49 years old, 21% (n=87) were aged 50-59 years old, and 18.8% (n=78) were aged >59 years old (mean age 45.6±14.0). MT (>2 mm) was observed in 76.4% (n=317) of participants. The mean mucosal thickness was 5.5 ± 5.4 mm (range=0-32 mm).

The mean ABL was 33.9% ± 11.0 (range: 16%-81.5%). Twenty percent of participants (n=83) had mild, 71.6% (n=297) had moderate, and 8.4% (n=35) had severe ABL. Participants in the severe bone loss category were combined with those in the moderate category because this category was too small to provide a meaningful analysis.

The prevalence of MT was significantly higher among male participants (83.5%) than among female participants (69.8%) (P=0.001). The prevalence of MT was significantly higher in the participants with increased ABL severity (P=0.03). No significant association was found between MT and other background characteristics, including age, nationality, and the presence of medical conditions.

Table 2 shows the multivariate association between ABL and MT. This association was analyzed using a logistic regression model. The final model was adjusted for age and sex. The overall model was statistically significant (P < 0.01).

**Table 2:** Multivariate logistic regression model for the association between ABL and MT *Statistically significant; OR: Odds ratio; 95% CI: 95% Confidence Interval; Ref: Reference group

	MT		
Variables	OR	95% CI	P-value
ABL			
Mild	Ref	Ref	0.04*
Moderate/severe	1.8	1.04-3.2	
Gender			
Male	2.2	1.4-3.6	0.001*
Female	Ref	Ref	
Age			
≤35 years	Ref	Ref	0.5
36–49 years	1.3	0.7-2.5	
50–59 years	0.9	0.5-1.7	
≥60 years	0.8	0.4-1.7	

ABL was found to be a statistically significant determinant of MT among the study participants, holding the other variables in the model constant. The model indicated that participants with moderate/severe ABL had a significantly higher risk of MT with an OR of 1.8 as compared with those with mild ABL (95% CI: 1.04-3.2).

Sex was found to be a statistically significant determinant of MT in this sample, holding other variables in the model constant. The male group had an increased risk of MT compared to the female group (OR =2.2, 95% C.I.: 1.4-3.6).

## Discussion

In this cross-sectional study, we aimed to define and assess the relationship between periodontitis and maxillary sinusitis in adult patients with periodontitis using CBCT images of at least one maxillary sinus. Using the collected data, we found that the mean age of patients who have periodontitis is 45.6 (±14.0), which is consistent with recent epidemiological studies [39]. Most of the observed cases (317) showed abnormal MT (>2 mm). A study by Ren et al. included 221 individuals with periodontal disease who underwent a cross-sectional CBCT scan. They reported that MT was found in 103 (48.9%) of the 221 patients examined with periodontal disease. When comparing MT with the severity of ABL, a distribution of 14.5%, 29.5%, and 87.9% was observed in patients, representing mild, moderate, and severe ABL, respectively. These results demonstrated a significant increase in MT as ABL increased [30].

According to our study, MT and ABL were significantly associated, which is consistent with previous studies that found that bone loss has a significant association with the degree of MT [11,16,25,28,30]. A multivariate logistic regression test revealed that participants with moderate or severe ABL demonstrated a statistically significant increased risk of MT, with an OR of 1.8 compared to patients with mild ABL. This finding is consistent with previous studies [25,28,30]. Our findings highlight the influence of ABL on maxillary sinus thickness. Compared to patients who had mild ABL, those who had increased bone loss severity had a 1.8 times higher chance of developing maxillary MT.

MT may be affected by periodontitis due to localized inflammation as bacterial invasion occurs. The presence of microporosities on the floor of the alveolar bone is basic proof of bacteria spreading and invading the maxillary sinus. Therefore, the condition of the maxillary sinus mucosa can be affected by periodontal infection [40]. In previous studies, periodontal pathogenic bacteria such as Fusobacterium nucleatum and Prevotella intermedia were found in a maxillary sinus lesion, supporting the idea that these microorganisms are able to reach the maxillary sinus through the microporosities in the alveolar bone. Thus, it can lead to the production of an inflammatory reaction in the maxillary sinus [41].

The average MT in our study was 5.5 mm, which corresponded with the previous study by Phothikhun et al., where they found the average MT to be 5.0 mm [25], while in contrast, the study by Apparaju et al. found an average of 3.43 mm of MT. Additionally, a study by Zhang et al. found the average MT to be 8.25 mm [13,28]. These results found in the literature can be explained by the sample size because the study, which was consistent with our results, had a sample size of 500 CBCT images, which approximates our sample size. On the other hand, studies that showed inconsistent results compared to ours had smaller sample sizes.

Regarding the sex factor we found in our study, MT was significantly higher in males, which was similar to the results of previous studies [15,25,30]. However, in the study conducted by Brüllmann et al., they reported no association between gender and MT [17]. The average age found in our sample, which included only periodontitis patients, was 45.6 (±14.0), which we found inconsistent with the results of the study by Zhang et al., where they found the average to be 54.1 (±11.8) given the fact that their sample was only periodontitis cases as well [28].

This study highlighted that moderate/severe periodontitis increases the risk of MT. Therefore, periodontal therapy should be initiated prior to maxillary sinus surgery to reduce mucosal membrane inflammation. In the study by Lathiya et al., where they compared two groups of patients with MT and chronic periodontitis, after treating one group with surgical periodontal therapy while controlling the other, a reduction in MT was seen in the group that received periodontal therapy [42]. Finally, to provide the foundation for appropriate clinical management, further research on the nature and causes of these anomalies is required.

The study had a few limitations. Our study had a cross-sectional design; thus, temporality cannot be evaluated, and we do not know which variable occurred first: loss of bone or thickening of mucosa. This is not a clinical study; hence, we could not examine the symptoms of patients and assess the severity of diagnosis properly, and we could not record medical history accurately because the data were taken from the Salud software system and are subject to inaccuracy.

## Conclusions

In conclusion, the study suggests that there is a significant association between periodontitis and maxillary sinusitis. Thus, it is crucial to keep in mind the health status of periodontium in the diagnosis and management of maxillary sinusitis patients. Additionally, it emphasizes the importance of a comprehensive dental examination, including a thorough assessment of periodontal structures when evaluating individuals with suspected maxillary sinusitis. Moreover, further clinical studies are warranted to better understand the precise relationship between periodontal and maxillary sinus structures and to develop optimal treatment strategies for patients presenting with both periodontal disease and maxillary sinusitis.
